# Adhesin RadD: the secret weapon of *Fusobacterium nucleatum*

**DOI:** 10.1080/19490976.2024.2426617

**Published:** 2024-11-09

**Authors:** Dingjiacheng Jia, Shujie Chen

**Affiliations:** a Department of Gastroenterology, Second Affiliated Hospital of Zhejiang University School of Medicine, Hangzhou, Zhejiang Province, China; b Institution of Gastroenterology, Zhejiang University, Hangzhou, Zhejiang Province, China; c Department of Gastroenterology, Sir Run Run Shaw Hospital, Zhejiang University, Hangzhou, Zhejiang Province, China

**Keywords:** Adhesin, CD147, colorectal cancer, fusobacterium nucleatum, gut microbiota, RadD, virulence proteins

## Abstract

*Fusobacterium nucleatum* can promote colorectal cancer (CRC) development through a variety of virulence proteins. Zhang et al. recently identified an adhesin RadD, for *Fusobacterium nucleatum* adhesion to CRC. Targeting the interaction between RadD and CD147 will provide a new strategy for CRC treatment. This Commentary and View not only summarizes the research highlights but also discusses the possibility of targeted clearance of *Fusobacterium nucleatum* in clinical applications.

## *Fusobacterium nucleatum* promotes colorectal cancer development through several adhesin

**Figure 1.**
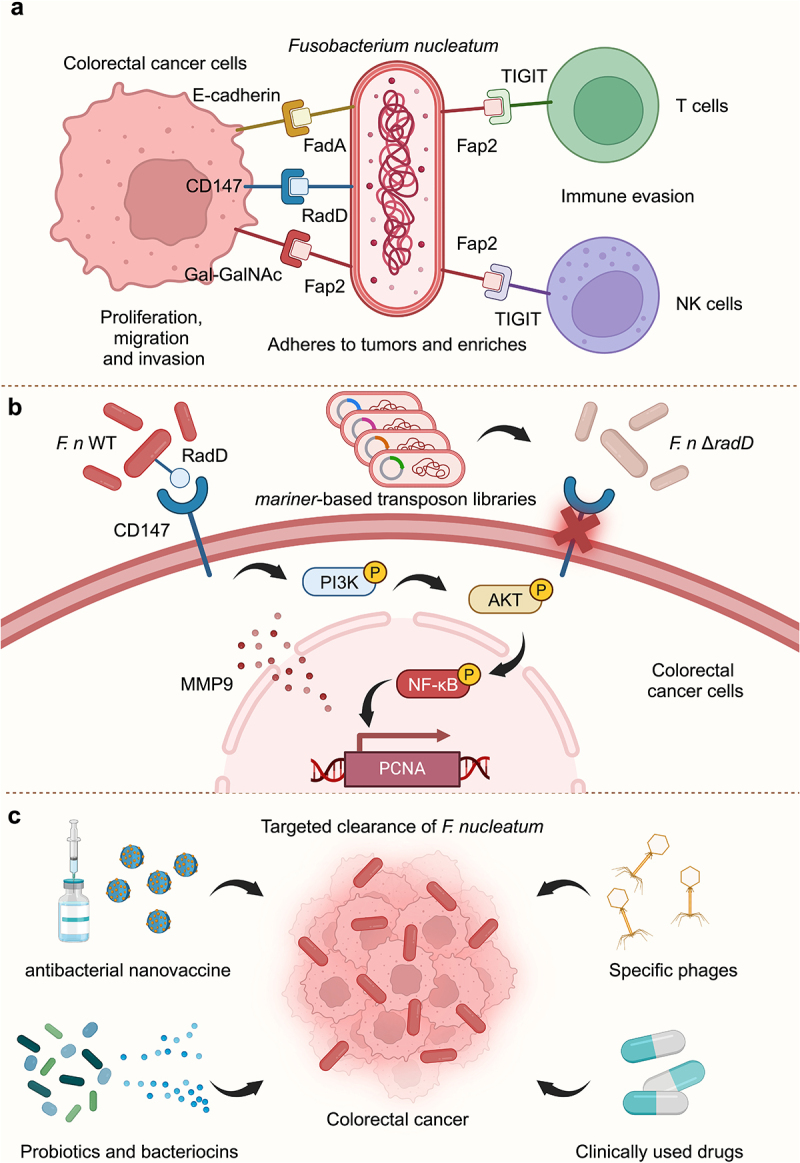
Adhesin RadD: the secret weapon of *Fusobacterium nucleatum*. (a) *Fusobacterium nucleatum* promotes colorectal cancer development through several adhesin. FadA binds to E-cadherin; Fap2 binds to gal-GalNAc of tumor cells and TIGIT inhibitory receptors on the surface of T cells and NK cells to initiate immune evasion; Zhang et al. found that RadD could bind to CD147 receptor. (b) Zhang et al. successfully screened for a novel adhesin of *Fusobacterium nucleatum*, RadD, based on the *mariner*-based transposon libraries. RadD could bind to CD147 and activate the downstream PI3K-AKT-NF-κB-MMP9 signaling pathway to promote colorectal cancer proliferation. (c) in the future, material-modified nanovaccine, probiotic-derived bacteriocins, specific phages, and clinical drugs can be used to target the elimination of *Fusobacterium nucleatum*. Abbreviations: TIGIT, T cell immunoglobulin and ITIM domain; NK cells, natural killer cells; WT, wild type; MMP, matrix metalloproteinases. Figure was created with BioRender.com.

*Fusobacterium nucleatum* is a common colonizing bacterium in the human oral cavity but is enriched in colorectal cancer (CRC) tissues and influences CRC development.^1^ Similar to other pathogenic bacteria, *F. nucleatum* could express a variety of surface adhesion proteins to effectively adhere to tumors. FadA and Fap2 are two known virulence proteins of *F. nucleatum*. FadA binds to the E-cadherin on the host colon epithelial cells and promotes colorectal carcinogenesis through activation of the Wnt-β-catenin signaling pathway, whereas Fap2 binds to Gal-GalNAc on the surface of CRC cells and mediates the enrichment of *F. nucleatum*. Fap2 can also bind to T cell immunoglobulin and ITIM domain (TIGIT), an inhibitory receptor on the surface of T cells and natural killer (NK) cells to initiate immune evasion. Notably, the double knockout strains (*F. n* ∆*fap2 fadA*) still have adhesion potential, suggesting that *F. nucleatum* may contain other adhesion proteins.^2^ In September 2024, Zhang et al. found that *F. nucleatum* can promote CRC development through another adhesin RadD^3^ (Figure 1(a)).

## Adhesin RadD promotes colorectal cancer proliferation by binding to CD147

Zhang et al. first constructed a series of mutant *F. nucleatum* strains using the *mariner*-based transposon libraries. When these mutant strains were co-cultured with CRC cells, three strains showed a significant reduction in their ability to adhere to CRC cells. Interestingly, all three bacterial transposons were inserted into the *radD* gene encoding the RadD protein. As an adhesin, RadD is mainly involved in microbial interactions between *F. nucleatum* and a variety of Gram-positive bacteria or fungi to promote biofilm formation.^4^ To verify the potential effect of the RadD protein on CRC, Zhang et al. constructed the mutant strain (*F. n* ∆*radD*) and the complementation strain (*F. n* ∆*radD/radD*). *In vitro* co-culture experiments revealed that *F. n* ∆*radD* strain reduced the adhesion ability, while the *F. n* ∆*radD/radD* strain rescued this phenotype. Once attached to the surface of CRC cells, *F. nucleatum* could promote tumor cell proliferation in a variety of ways. The authors further found that RadD could also mediate the pro-proliferative effects of *F. nucleatum*, and gavage of *F. n* ∆*radD* did not promote CRC growth.

To explore the potential value of RadD in clinical practice, Zhang et al. found that the expression of *radD* in CRC tissues was significantly higher than that in non-cancerous tissues. As an independent risk factor, the expression level of *radD* was closely associated with tumor size, clinical stages, CRC aggressiveness, and recurrence risk. Mechanistically, Zhang et al. found that adhesion of *F. nucleatum* to normal intestinal epithelial cells was not regulated by RadD, suggesting that RadD may bind to a receptor specifically expressed on the surface of CRC cells. Using a combination of genetic and proteomic approaches, Zhang et al. targeted CD147, whose activation could promote the secretion of matrix metalloproteinases (MMPs) involved in CRC proliferation, migration, and invasion. RNA sequencing and mouse experiments revealed that RadD binding to CD147 could activate the downstream PI3K-AKT-NF-κB-MMP9 signaling axis. Whereas the administration of CD147 antibody significantly inhibited *F. nucleatum* adhesion and attenuated the activation of the pro-oncogenic cascade signaling pathway, thus effectively reducing CRC burden.

### Key study findings and perspective

Overall, Zhang et al. identified RadD as a novel virulence protein for *F. nucleatum* adhesion to CRC and revealed its mechanism in CRC development (Figure 1(b)). There are some highlights of this study that are worthwhile for future related studies. First, the authors used a library system based on transposons. Unlike *E. coli*, which has many expression vectors for modification, it is not easy to achieve homologous recombination in non-model strains, but this study by Zhang et al. provides a way. By using transposons to make random mutations in the genome, we can quickly achieve knockout of the target bacteria.^5^ However, it should be noted that the strain modification needs to be followed by batch screening if it is targeting a specific gene. Second, RadD is used by *F. nucleatum* to interact with a variety of cell types such as tumor cells and other microorganisms. In this study, *F. nucleatum* exerts its pro-carcinogenic effects through RadD and CD147 receptor binding in CRC cells. Since *F. nucleatum* may also broadly affect the immune microenvironment of tumors, future studies may attempt to explore the potential effects of RadD on immune cells. Is there a difference of CD147 expression in different immune cells? Can *F. nucleatum* escape immune surveillance by inhibiting CD147 expression on macrophages or dendritic cells? Furthermore, the CD147 molecule is noteworthy for future clinical applications. As a receptor for trefoil factor 3 (TFF3), CD147 is essential for mucosal restoration and CRC progression.^6^ In addition to *F. nucleatum*, several other human gastrointestinal pathogens have the ability to target CD147. Perhaps, the development of clinical CD147-specific antibodies will provide a new strategy for tumor therapy.

As a widely recognized cancer-promoting ‘bad’ bacterium, how to specifically kill *F. nucleatum* is still an outstanding question. Some recent research trends are noteworthy. Precise delivery using materials may be a good option. A recent study by Chen et al. has developed an emerging antibacterial nanovaccine that could eliminate *F. nucleatum* without affecting the intratumoral and gut microbiota.^7^ This nanovaccine could stimulate a stronger immune response due to the co-delivery of *F. nucleatum* membrane antigens and the highly immunostimulatory adjuvant to antigen-presenting cells. In addition, screening of microbial metabolites with narrow-spectrum antimicrobial activity may mitigate the overall impact on the host intestinal microecology. *Streptococcus salivarius* strain DPC6993, which exhibits specific antimicrobial activity against *F. nucleatum*, has recently been successfully isolated from the feces of healthy donors.^8^ Its genome encodes two bacteriocins, salivaricin A5 and salivaricin B, which inhibit the growth of *F. nucleatum in vitro* and in a colon fermentation model. And Zheng et al. applied *F. nucleatum*-specific phage isolated from human saliva also prolonged the survival of mice with CRC.^9^ It is worth mentioning that some clinically used drugs such as metformin and aspirin were also found to have the effect of inhibiting the growth of *F. nucleatum*^10^ (Figure 1(c)). Although many potential ways are targeting *F. nucleatum* through material modification, application of probiotics or phages, or drug intervention, there’s still a long way to set into clinical applications.
